# Supplementary material for: Social contact patterns and implications for infectious disease transmission – a systematic review and meta-analysis of contact surveys
Source: eLife. 2021 Nov 25;10:e70294. doi: 10.7554/eLife.70294 (PMC8765757; doi:10.7554/eLife.70294)
Supplement: Supplementary file 1. [file elife-70294-supp1.docx]

***Supplementary file 1: Search string***

| 1 | (social contact* or contact pattern* or contact network* or contact mixing or contact survey* or contact data or contact rate* or contact matri* or contact parameter* or physical contact* or social mixing or mixing behavio* or mixing pattern* or mixing matri* or assortative mixing or disassortative mixing or mixing parameter*).af. |
| --- | --- |
| 2 | (**LIC** or **low-income*** or **low income*** or **low- income*** or **developing countr*** or Afghanistan or Guinea-Bissau or Sierra Leone or Benin or Haiti or Somalia or Burkina Faso or Korea or Sudan or Burundi or Liberia or Syria* or Central African Republic or CAR or Madagascar or Tajikistan or Chad or Malawi or Tanzania or Congo or DRC or Mali or Togo or Eritrea or Mozambique or Uganda or Ethiopia or Nepal or Yemen or Gambia or Niger or Guinea or Rwanda or LMIC or Angola or India or Papua New Guinea or Bangladesh or Indonesia or Philippines or Bhutan or Kenya or (Sao Tome and Principe) or Bolivia or Kiribati or Senegal or Cabo Verde or Kyrgyz Republic or Solomon Islands or Cambodia or Laos or Cameroon or Lesotho or Timor-Leste or Comoros or Mauritania or Tunisia or Micronesia or Ukraine or Cote d’Ivoire or Moldova or Uzbekistan or Djibouti or Mongolia or Vanuatu or Egypt or Morocco or Vietnam or El Salvador or Myanmar or “West Bank and Gaza” or Eswatini or Nicaragua or Zambia or Ghana or Nigeria or Zimbabwe or Honduras or Pakistan or **middle income*** or **middle-income*** or **middle- income** or **UMIC** or Albania or Algeria or American Samoa or Argentina or Armenia or Azerbaijan or Belarus or Belize or (Bosnia and Herzegovina) or Botswana or Brazil or Bulgaria or China or Colombia or Costa Rica or Cuba or Dominica or Dominican Republic or Guinea or Ecuador or Fiji or Gabon or Georgia or Grenada or Guatemala or Guyana or Iran or Iraq or Jamaica or Jordan or Kazakhstan or Kosovo or Lebanon or Libya or Malaysia or Maldives or Marshall Islands or Mauritius or Mexico or Montenegro or Namibia or Nauru or North Macedonia or Paraguay or Peru or Romania or Russia* or Samoa or Serbia or Sri Lanka or South Africa or Lucia or (Vincent and the Grenadines) or Suriname or Thailand or Tonga or Turkey or Turkmenistan or Tuvalu or Venezuela).af. |
| 3 | 1 and 2 |
