# Supplementary material for: Social contact patterns and implications for infectious disease transmission – a systematic review and meta-analysis of contact surveys
Source: eLife. 2021 Nov 25;10:e70294. doi: 10.7554/eLife.70294 (PMC8765757; doi:10.7554/eLife.70294)
Supplement: Supplementary file 4. [file elife-70294-supp4.docx]

***Supplementary file 4. Study additional Information and data assumptions***

| **Study** | **Additional data information and assumptions** | **Data source** |
| --- | --- | --- |
| **China**(Read et al., 2014) | The survey was carried out between 2009 and 2010, with interviewer led questionnaires in which study participants reported all the people they encountered the previous day. Parents were interviewed on behalf of children deemed too young to provide reliable information for both the individual participant questionnaire and the contact diary. The study did not differentiate between unique contacts across contact events, and sometimes the same individual may appear in multiple events reported by a participant. The total number of contacts reported by the participant was obtained using variable “**c.all”** in the original publication data. The number of non-physical contacts was derived by subtracting the number of reported physical contacts from the total number of contacts. | Supplementary data from publication |
| **China**(Zhang et al., 2020) | The survey was carried out between December 2017 and May 2018 in Shanghai. Participants were requested to record each contact they made on the assigned day (during the 24 hours before going to bed). The study used both prospectively completed paper diaries and retrospectively collected telephone interview surveys.  A household size of 0 was assumed to be an error and was set to missing.  The questionnaire allowed participants to report a maximum number of 40 individual contacts. Participants were asked to report group contacts (Zenodo variable “**group_n”**), defined as a contact with a group of at least 20 individuals. Additionally, they were asked to include the number of contacts they left out (Zenodo variable “**num_left_out**”). Contacts entered in “**group_n”** and “**num_left_out”** were mutually exclusive and both were added to the individual contacts in the main analysis (and excluded in the sensitivity analysis) | Zenodo |
| **European**(Mossong et al., 2008) | The surveys were conducted between May 2005 and September 2006 and were implemented by different commercial companies or public health institutes in eight European countries. Participants were assigned a random day of the week to record every person they had contact with between 5 a.m. and 5 a.m. the following morning. Participants were instructed to record contacted individuals only once in the diary. Diaries for young children were filled in by a parent or guardian on their behalf. The maximum number of contact entries in the diary varied between 29 (UK) and 90 (Belgium). Participants were instructed to not record professional contacts in the diary (eg. with clients) for 4 of the countries if they were above a certain number (Belgium if >20 professional contacts; Germany, Finland and Netherlands if >10 professional contacts). This instruction may have led to some underreporting of contact frequencies in these countries. The number of these additional professional contacts in addition to the estimated number of contacts left out of the survey, which was reported for some surveys (e.g UK), are not available in the public domain (and hence were not used). For further differences in the way surveys were conducted, refer to the supplementary material of the original publication.  In the present study, we obtained employment and student status for participants who reported an occupation (variable “**part_occupation**”). Participants were considered as being in employment if they reported “working”) and as being a student if they reported “in fulltime or further education”. | Zenodo |
| **Fiji**(Neal et al., 2020) | A social contact questionnaire was administered between August and November 2015. An experienced study nurse interviewed caregivers by telephone and recorded contact details for them and each of their child participants over the previous 24 h (following a nasopharyngeal carriage survey).  Data on contacts made at work were unavailable as this study focused on children and their caregivers. Participants for which an occupation was reported were considered in employment, unless they were one of the following: “Housewife”, “Retired”, “Unemployed”, children under 6 years, or “secondary/college student” (considered as not being in employment). If a participant was not reported as a student and was in employment, they were considered as not being a student. | Data received by the authors of the original publication |
| **Hong Kong**(Kwok et al., 2014) | An interviewer-led social-contact questionnaire was carried out between 2009 and 2010. An estimated range for total contacts was reported by the respondents and was available from the original dataset (variables **contactalltotalmax** and **contactalltotalmin**). For the present study, the number of daily contacts was calculated as the mean of **contactalltotalmax** and **contactalltotalmin,** rounded. Similarly, the total number of physical contacts was estimated as the mean of **contactclosemin** and **contactclosemax**, rounded. The same method was used for estimating the number of contacts of a particular duration (e.g. total number of contacts made that lasted over an hour).  Participants reporting an occupation were considered to be in employment unless they reported “housewife”, “retired”, “student”, “economically inactive person” or “maid” (considered as not being in employment). If a participant did not report being a student, and they reported an occupation, they were considered as not being a student. | Supplementary data from publication, with additional data received from authors on participant gender household size, day type, student status and employment. |
| **Hong Kong**(Kwok et al., 2018) | The surveys took place between May 2012 and September 2013. Only data from the first wave were used (N=1,066). Participants would be interviewed about a randomly assigned day within 4 days after the assigned reporting day. For a single contact made multiple times during the same day, multiple locations could be reported.  The coding for occupation was identical to the Kwok 2014 study, and the same procedures were used as explained above to code employment and student status.  The number of contacts was reported for individual locations ie. home, school, work, and other (“eat”, “play”, “shop”, “transport”, “meet”, “other”). The total number of daily contacts was obtained from original variable “**ncontacttotal**”. The number of non-physical contacts was derived by subtracting the number of reported physical contacts from the total number of contacts. | Supplementary data from publication, with additional data received from authors on household size, contact location, student status and employment. |
| **Hong Kong**(Leung et al., 2017) | The contact survey took place between 2015 and 2016. A total of 430 participants filled in a paper diary and 719 filled in an online questionnaire. For a single contact made multiple times during the same day, multiple locations could be reported.  There were cases where the participant had not reported all the contacts encountered on the assigned day and these are recorded in a variable named “**num_left_out**” (estimated number of contacts left out). These were included in the main analysis and excluded in a sensitivity analysis.  Participants reporting (in original variable **work_role**) being an “employee”, an “employer”, or “self-employed” were considered as being in employment and those reporting being “students”, “homemakers” or “retired” were considered as not being in employment. “others”, “don’t know” or “unwilling to answer” were coded as missing for both student and employment statuses. If a participant did not report being a student, and they reported an occupation, they were considered as not being a student. | Zenodo |
| **India**(Kumar et al., 2018) | Interviews took place between October 20, 2015 and February 29, 2016. All individuals in each household were interviewed and asked about their contacts in the past 24 hours. A caregiver responded for children five years old or younger, whereas children 6–10 years old responded in the presence of a caregiver. For a single contact made multiple times during the same day, multiple locations could be reported. Respondents could report an encounter with multiple individuals as a “group” contact. These were included in the main analysis and excluded in a sensitivity analysis.  Participants were asked "Are you enrolled in school OR college? (yes=student, no=not a student) and “Are you employed outside the home?” (yes=in employment, no=not in employment). Additional data on occupation were used to help define employment status. If participants reported an occupation they were considered in employment unless they reported being “Dependents (Still studying)” , “Aged individuals”, “Retired”, “House Wife”, “Girls not studying but doing household chores” or “Unemployed”, even if they had answered “no” to “Are you employed outside the home?”. If the answer to this question was missing, and participants reported any of the above in the occupation field, they were considered as not being in employment. Similarly, if occupation was recorded as “Dependents (Still studying)” and the answer to "Are you enrolled in school OR college?” was missing, they were considered as students. | OSF, and additional data received from authors on household size and occupation |
| **Kenya**(Kiti et al., 2014) | The study took place over the period 17th August 2011 to 31st January 2012. For participants under 10 years old who were unable to read and write, a “shadow” was asked to report their contacts. Participants were expected to keep the diary for a day, defined as the period between first waking and going to bed for the night. Each contact was recorded only once in the diary during the day of study, and repeat encounters were recorded as tallies. Participant occupation was recorded by original publication variable “**part_job**”. If participants reported an occupation they were considered in employment unless they reported being a “Student , “Pre-School”, “Retired” or “Unemployed” (considered not in employment). | Supplementary data from publication, with the addition of exact participant age received from authors. |
| **Peru**(Grijalva et al., 2015) | The contact survey was carried out from May 2009 through September 2011 and participants reported the number of contacts made over a 24 hour period (5am to 5 am). Contact information for children younger than 10 years was provided by the parents. For a single contact made multiple times during the same day, multiple locations could be reported. Original variables **contd005** and **contd005s** that record the participant’s occupation were used to classify employment. For those who reported an occupation, they were assumed to be “employed” unless they reported the following in contd005 or contd005s: "ESTUDIANTE", “ESTUDIASUPERIOR", "En la escuela", "Su casa" (all 3 of which were coded as student), "ABUELAPATERNA", "ENELJARDIN", "JARDIN", "JARDINLIBRE", "WAWASI" (these were coded as “not in employment”). Participants who reported an occupation, were coded as not being a student. | Zenodo |
| **Russia**(Ajelli and Litvinova, 2017) | The survey was conducted between January 28, 2016 and February 26, 2016. The data which were collected during as school closure reactive to pathogen transmission (variable **period**) were excluded (ie. period== “school_closure”).  For a single contact made multiple times during the same day, multiple locations could be reported.  All surveys were reporting for weekdays.  In the original data the variable activity_status reported whether a participant was a “Student”, a “Worker” or “Inactive”. “Student” was assumed to be a student and not in employment, “Worker” was assumed to be in employment and not a student, and “Inactive” was assumed to be neither a student nor in employment.  For workers who have large number of contacts at work (such as cashiers of supermarkets, waitresses), study participants were asked to provide an estimate of the number of people that they have contacted at work, referred to as additional professional contacts (found in original variable “**number_of_other_work_contacts”** in Zenodo). These contacts were included in the main analysis and excluded in a sensitivity analysis. | Zenodo and additional data received by authors on 52 additional diaries |
| **S Africa**(Wood et al., 2012) | The contact survey was carried out over a period of 4 months in 2010 and participants reported the number of contacts made over a 24 hour period (5am to 5 am). For participants under 11 years of age, parents/guardians completed the diary survey together with the child. Participants were asked to report each contact once, but also recorded whether it was the first time each close contact had been met within the 24-hour period. For a single contact made multiple times during the same day, multiple locations could be reported.  Data received in the required format, so no data assumptions were made. | Data received by the authors of the original publication |
| **Senegal**(Potter et al., 2019) | Data were collected between August 1, 2009 to February 1, 2010 and respondents reported the number of people they contacted in their own compound on both the morning (AM) and the evening (PM) of the survey day. Next, they were asked whether they had visited a list of twelve geographic locations on the survey day, including up to five (non-home) compounds, a field, market, mosque/church, and others. For each location that was visited, respondents reported the time of day (AM, PM, or both) and the number of people contacted in that location. The same information was collected for the preceding two days. For children too young to respond to the survey, the questions were answered by a parent or guardian.  In the current meta-analysis study, only contacts reported for the day before the survey (ie. “yesterday”) were considered, so that is comparable with other studies. Additionally, a complete case analysis was used that ignores those who have missing number of contacts made outside or inside the home for any of the locations (variables **spokenumberyesterday1**,…. , **spokenumberyesterday12**, **contactsnumberyesterdayam**, **contactsnumberyesterdaypm).** A complete case analysis reduces the data to a subset of 1,417, which were used. A limitation of this approach is that it may underestimate the number of contacts as those who reported having visited a location but did not report the contact number were not used in the analysis.  No “work” location was recorded, and the total number of contacts was calculated as the sum of contacts made by an individual in their own compound and in other locations.  The total number of contacts made by a participant in a day was calculated as the sum of contacts made by a single participant in the morning and in the afternoon (e.g. number of contacts made at home =sum of **contactsnumberyesterdayam** and **contactsnumberyesterdaypm** variables in the OSF data.  **For further details on methodology and citing these data, please refer to the original publication.** | OSF |
| **Thailand**(Mahikul et al., 2020) | The survey was conducted in various workplaces in Pathum Thani, Thailand, between September and November 2015 and participants were asked to record their contacts in the past 24 hrs. For a single contact made multiple times during the same day, multiple locations could be reported. Variable **Occupation** in the original study was recorded as : “Agricultural worker”, “general labor” or “merchant” (all 3 of which were coded as being in employment), “other”, “NA”, both coded as missing. Contact age was available in broad age groups: <5, 5 to 15, 15 to 40, >40. | Zenodo |
| **Thailand**(Stein et al., 2014) | Online surveys were used to collect contact network patterns in Thailand between November 2012 and May 2013. Participants were asked to record the number of contacts they had the day before.  Household size was available from the pilot study and the remaining data were obtained from the second publication.  The total number of contacts were obtained from variable **degreeyourspace** in the original data. Two observations with extremely high number of contacts (2,233 and 4,456 contacts made within one day were considered as data entry errors and were set to missing. An additional 36 participants had missing number of contacts and were excluded from the analysis. | Data from two publications (pilot and follow-up study) both available on figshare. |
| **Uganda**(le Polain de Waroux et al., 2018) | The survey took place between January and March 2014 and participants were asked about their social contacts in the 24 h preceding the survey. For children < 5 years, parents were asked about their child’s encounters. Encounters reported with the same individual in different settings counted as one contact only.  Participants were asked to select their occupation (variable **occupation** in supplementary file of published study). Those reported as “school college university student” were coded as students. The following occupations were considered to be in employment: agriculture, manual worker, office worker, shop worker. If one of these occupations were selected, participants were coded as not being students. If participants were recorded in the **occupation** variable as “unemployed” or “housewife” or “pre-school child” or “retired” or “school college university student”, they were considered as not being in employment. | Publication data supplement and additional data received from authors on gender, exact age and household size. |
| **Vietnam**(Horby et al., 2011) | The survey took place in 2007, and subjects recorded the details of each contact made on the day preceding the interview. If an individual was contacted multiple times during the day, the individual was recorded only once but the total time spent with that person during the day was entered. For a single contact made multiple times during the same day, multiple locations could be reported.  If a participant reported being a “student” when listing their occupation variable (variable **part_occupation_detail** in Zenodo file), they were reported as being a student. If an occupation was reported, participants were coded as not being students. Those reporting any occupation were assumed to be in employment and for those reporting “unemployed” or “student” were assumed not to be in employment. | Zenodo |
| **Zambia and South Africa**(Dodd et al., 2015) | Interviews took place in February and March 2011 in Zambia and in May and July 2011 in South Africa. Interviewees were asked to report contacts that occurred in the 24 hours preceding the midnight before the interview.  For determining employment status, the following question was asked: “How have you contributed to household living during the past year?” (variables **q56_job_0** to **q56_job_7** in Zenodo). Participants with the following answers were considered as being in employment: “working own land”, “occasional/seasonal employment”, “employed” or “own business”. Participants who only reported “No contribution”, “Housewife-home-maker” or “Welfare grant” or “student” were considered as not being in employment. Participants who did not report being a “student” were considered as not being a student, only if any information on employment was recorded. | Zenodo |
| **Zimbabwe**(Melegaro et al., 2017) | Data were collected from March 2013 to August 2013 (which included a school holiday closure from March 28th to May 6^th^). Multiple contacts with the same individual were reported only once per day. For illiterate adults and children < 10 years, a designated “shadow” filled in the questionnaire on behalf of the study participant.  Contacts made by individuals were reported for two consecutive days. For the present study, only the contacts made within one day of the survey were used (i.e variable **studyDay**==2 using the **contact_extra.csv** file uploaded on Zenodo.  The employment status of the majority of participants (81%) was unknown. Sector of employment was recorded as: teacher, office worker, agriculture/fishing, retail, casual labour, retired, unemployed or “other” (variable “**work_sector**” in Zenodo file). For “other” work sectors, participants were asked to report their occupation (variable “**work_sector_detail”** in Zenodo file, but this was not filled in by most, and was recorded in this study as missing. | Zenodo |
| **General assumptions for all studies** | For the methodology, if a study employed both a diary-based method AND an interview at the end of the day, then methodology was considered as “Diary”. Structured questionnaires filled in during an interview retrospectively were considered as “Interview”.  Participants reporting a contact at work who have missing employment status were assumed to be employed.  Participants reporting a contact at school who have a missing student status were assumed to be students (for ages <=18). Entries for children aged<10 which were recorded as “employed” or for which a number of contacts at work was given, were set to missing.  Contact duration was categorized into <1hr and 1hr+ to utilize all data on contact duration.  For contact-level datasets, the number of total contacts per participant was calculated as the sum across contact rows for a given location (tot_home, tot_school, tot_work, tot_other). For a contact where “False” was recorded for all locations, location was coded as missing.  For the total number of contacts per participant per group (eg duration <1hr), the sum of contacts was calculated for each participant, unless all were missing. | NA |
