# Supplementary material for: Social contact patterns and implications for infectious disease transmission – a systematic review and meta-analysis of contact surveys
Source: eLife. 2021 Nov 25;10:e70294. doi: 10.7554/eLife.70294 (PMC8765757; doi:10.7554/eLife.70294)
Supplement: Supplementary file 5. [file elife-70294-supp5.docx]

**Supplementary file 5. Data dictionary for participant-level data**

| **Variable name** | **Description** |
| --- | --- |
| study | Survey country and first author of original publication |
| income | Country-level income group, as defined by the World Bank (LIC/LMIC, UMIC, HIC) |
| method | Survey methodology (Diary, Interview). “Interview” also includes retrospectively reported phone/online surveys. |
| part_gender | Participant gender (Female/Male |
| part_age | Participant age |
| age3cat | Participant age group (<15, 15 to <65, 65+) |
| hh_size | Household size |
| student | Student status (0=no, 1=yes) |
| employment | Employment status (0=no, 1=yes) |
| weekday | Survey day type (0=weekend, 1=weekday) |
| tot_contacts | Total number of daily contacts made by a participant. This includes additional contacts, including additional work contacts, group contacts and number of contacts left out. This variable is used in the main analysis. |
| tot_contacts_no_add | Total number of daily contacts made by a participant. This excludes additional contacts, such as additional work contacts, group contacts and number of contacts left out. This variable is used in a sensitivity analysis. |
| tot_phys | Total number of contacts made by a participant that were physical |
| tot_nonphys | Total number of contacts made by a participant that were not physical |
| tot_dur_under_1hr | Total number of contacts made by a participant which lasted under 1 hour |
| tot_dur_1hr_plus | Total number of contacts made by a participant which lasted an hour or longer |
| tot_home | The number of contacts made by a participant at home |
| tot_school | The number of contacts made by a participant at school |
| tot_work | The number of contacts made by a participant at work including additional work contacts (main analysis) |
| tot_work_no_additional | The number of contacts made by a participant at work without the inclusion of additional work contacts (sensitivity analysis) |
| tot_other | The number of contacts made by a participant at other locations |
| tot_miss | The number of contacts made by a participant with a missing location |
| prop_home | Proportion of contacts that occurred at home, among those with a known location |
| prop_school | Proportion of contacts that occurred at school, among those with a known location |
| prop_work | Proportion of contacts that occurred at work, among those with a known location |
| prop_other | Proportion of contacts that occurred at other locations, among those with a known location |
| prop_cont_male | Proportion of contacts that are male |
| prop_cont_female | Proportion of contacts that are female |
| prop_cont_age1  prop_cont_age2  prop_cont_age3 | Proportion of a participant’s contacts that belong to each of the 3 broad age groups (group 1= children aged 0 to 12-15; group 2= younger adults aged 13-16 to 40-49; group 3=older adults aged 41-50 or over)  Contact age was given as an exact age (green) or an estimated range or age group (yellow) and was categorized into three broad age groups. A total of 5,724 contacts out of 269,662 with available age information, but where the age range given was overlapping across the category bounds, were excluded in the assortativity analysis   \|  \| **Children 0 to 12-15** \| **Younger adults (13-16 to 40-49)** \| **Older adults (41-50 to max)** \| \| --- \| --- \| --- \| --- \| \| **European, Mossong** \| 0 to <15 \| 15 to <45 \| 45+ \| \| **China, Zhang** \| 0 to <15 \| 15 to <45 \| 45+ \| \| **Hong Kong, Leung** \| 0 to <15 \| 15 to <45 \| 45+ \| \| **India, Kumar** \| 0 to <15 \| 15 to <45 \| 45+ \| \| **Kenya, Kiti** \| <1,1-5,6-15 \| 16-19,20-49 \| 50+ \| \| **Peru, Grijalva** \| 0 to <15 \| 15 to <45 \| 45+ \| \| **Russia, Ajelli** \| 0 to <15 \| 15 to <45 \| 45+ \| \| **South Africa, Wood** \| 0 to <15 \| 15 to <45 \| 45+ \| \| **Uganda, Le Polain** \| <2, 2-4, 5-9, 10-14 \| 15-24, 25-34, 35-44, \| 45-54, 55-64, 65+ \| \| **Vietnam, Horby** \| 0-5, 6-15 \| 16-25, 26-34, 35-49 \| 50-64, 65+ \| \| **Zambia, Dodd** \| 0-4, 5-12 \| 13-25, 26-45 \| 46+ \| \| **South Africa, Dodd** \| 0-4, 5-12 \| 13-25, 26-45 \| 46+ \| \| **Zimbabwe, Melegaro** \| 0 to <15 \| 15 to <45 \| 45+ \| \| **Fiji, Neal** \| 0 to <15 \| 15 to <45 \| 45+ \| \| **Thailand, Majikul** \| 0-4, 5-14 \| 15-40 \| 41+ \| |
