# Supplementary material for: Social contact patterns and implications for infectious disease transmission – a systematic review and meta-analysis of contact surveys
Source: eLife. 2021 Nov 25;10:e70294. doi: 10.7554/eLife.70294 (PMC8765757; doi:10.7554/eLife.70294)
Supplement: Supplementary file 6. [file elife-70294-supp6.docx]

***Supplementary file 6. Extraction table of study characteristics***

| **Income status** | **Author, Year** | **Country, Area/District** | **Method** | **N (participants)** | **N (Contacts)** | **Contact definition** | **Data type** |
| --- | --- | --- | --- | --- | --- | --- | --- |
|  |  |  |  |  |  |  |  |
| **LIC** | (le Polain de Waroux et al., 2018) | Uganda, Southwest Uganda, Sheema | Interview | 568 | 3,964 | Two-way conversational encounters lasting for ≥5 min | Contact-level |
|  |  |  |  |  |  |  |  |
| **LMIC** | (Kumar et al., 2018) | India, Haryana, Faridabad district | Face-to-face Interview | 2,943 | 79,374 | A face-to-face conversation within 3 feet | Contact-level |
|  | (Kiti et al., 2014) | Kenya, Kilifi | Diary-based | 568 | 10,042 | Direct physical contact involving skin-to-skin touch | Contact-level |
|  | (Potter et al., 2019) | Senegal, Niakhar | Face-to-face Interview | 1,417 | 27,930 | A face-to-face conversation | Participant-level |
|  | (Horby et al., 2011) | Vietnam, Red River Delta | Diary-based | 865 | 6,675 | Skin-to-skin contact or a face-to-face two-way conversation | Contact-level |
|  | (Dodd et al., 2015) | Zambia, multiple locations | Face-to-face Interview | 2,300 | 11,028 | Face-to-face conversation that was longer than a greeting and within an arm's reach. | Contact-level |
|  | (Melegaro et al., 2017) | Zimbabwe, Manicaland | Diary-based | 1,245 | 13,282 | Skin-to-skin contact or a face-to-face two-way conversation | Contact-level |
|  |  |  |  |  |  |  |  |
| **UMIC** | (Read et al., 2014) | China, Guangzhou | Face-to-face Interview | 1,821 | 33,789 | A face-to-face conversation or skin-on-skin touch | Participant-level |
|  | (Zhang et al., 2020) | China, Shanghai (multiple locations) | Both telephone interview and diary-based | 965 | 18,116 | Skin-to-skin contact or a face-to-face two-way conversation | Contact-level |
|  | (Huang et al., 2020) | China, Guangdong, Pearl River Delta | Face-to-face Interview | 5,818 | ~96,500-97,100 | A conversation with three or more words or physical contact. | Not available |
|  | (Watson et al., 2017) | Fiji, multiple locations across Central, Northern and Western divisions | Face-to-face Interview | 1,814 | 9,650 | Sharing a meal or a table (meal time contacts) or physical (skin-to-skin) contacts. | Not available |
|  | (Neal et al., 2020) | Fiji, Suva | Telephone Interview | 2,019 | 12,932 | Skin-to-skin contact or all other contact in the physical presence of another person | Contact-level |
|  | (Grijalva et al., 2015) | Peru,San Marcos, Cajamarca | Face-to-face Interview | 588 | 9,009 | Skin-to-skin contact or a face-to-face two-way conversation no further than 3m apart | Contact-level |
|  | (Ajelli and Litvinova, 2017) | Russia,Tomsk | Diary-based | 502 | 9,026 | A face-to-face two-way conversation of at least five words | Contact-level |
|  | (Dodd et al., 2015) | South Africa,  multiple locations | Face-to-face Interview | 1,276 | 6,694 | "close contact": face-to-face conversation that was longer than a greeting and within an arm's reach. | Contact-level |
|  | (Wood et al., 2012) | South Africa, Cape Town | Diary-based | 571 | 8,919 | physical touch or a face-to-face 2-way conversation with 3 or more words | Contact-level |
|  | (Mahikul et al., 2020) | Thailand, Pathum Thani | Diary-based | 369 | 8,356 | either skin-to-skin contact or a two-way conversation, approximately one meter apart | Contact-level |
|  | (Stein et al., 2014) | Thailand, Bangkok | Online survey | 219 | 12,812 | A person sitting or standing within arm's length of the participant for 30 seconds or longer | Participant-level |
|  | (Meeyai et al., 2015) | Thailand | Diary-based | NA | NA | Physical skin-to-skin contacts or a face-to-face two-way conversation | Not available |
|  | (Oguz et al., 2018) | Turkey, Ankara | Diary-based | 1,006 | 4,706 | physical skin-to-skin contacts or interaction in close proximity with three or more words directed to the infant | Not available |
|  |  |  |  |  |  |  |  |
| **HIC^*^** | (Mossong et al., 2008) | Belgium | Diary-based | 750 | 8,878 | Skin-to-skin contact or a face-to-face 2-way conversation with 3 or more words | Contact-level |
|  |  | Finland |  | 1,006 | 11,128 |  |  |
|  |  | Germany |  | 1,341 | 10,659 |  |  |
|  |  | Italy |  | 849 | 16,784 |  |  |
|  |  | Luxembourg |  | 1,051 | 18,352 |  |  |
|  |  | The Netherlands |  | 269 | 3,726 |  |  |
|  |  | Poland |  | 1,012 | 16,501 |  |  |
|  |  | United Kingdom |  | 1,012 | 11,876 |  |  |
|  | (Kwok et al., 2014) | Hong Kong | Interview | 762 | 13,980 | Face-to-face conversation or skin-on-skin contact | Participant-level |
|  | (Kwok et al., 2018) | Hong Kong | Interview | 1,066 | 13,696 | Face-to-face conversation or skin-on-skin contact | Participant-level |
|  | (Leung et al., 2017) | Hong Kong | Diary-based and online | 1,149 | 16,541 | Skin-to-skin contact or a face-to-face 2-way conversation with 3 or more words | Contact-level |

*studies conducted in HIC, were not part of the systematic review, but were used as a comparison in the individual participant meta-analysis
