# Supplementary material for: Social contact patterns and implications for infectious disease transmission – a systematic review and meta-analysis of contact surveys
Source: eLife. 2021 Nov 25;10:e70294. doi: 10.7554/eLife.70294 (PMC8765757; doi:10.7554/eLife.70294)
Supplement: Supplementary file 7. [file elife-70294-supp7.docx]

***Supplementary file 7. Data availability by study***

|  |  | **Outcomes** | | | |  | **Study/ participant characteristics** | | | | | | |  | **Contact characteristics** | | |
| --- | --- | --- | --- | --- | --- | --- | --- | --- | --- | --- | --- | --- | --- | --- | --- | --- | --- |
| **Study** | **Income** | **Total contacts** | **Contact location** | **Contact type (physical/ non-physical)** | **Contact duration** |  | **Age** | **Gender** | **Day type (weekend or weekday)** | **Household size** | **Student status** | **Employment status** | **Method (diary/ interview)** |  | **Gender** | **Age** |  |
| **China** (Read et al., 2014) | **UMIC** | ✓ | 🗶 | ✓ | ✓ |  | ✓ | ✓ | 🗶 | 🗶 | 🗶 | 🗶 | ✓ |  | 🗶 | 🗶 |  |
| **China** (Zhang et al., 2020) | **UMIC** | ✓ | ✓ | ✓ | ✓ |  | ✓ | ✓ | ✓ | ✓ | 🗶 | 🗶 | ✓ |  | ✓ | ✓ | (exact) |
| **European** (Mossong et al., 2008) | **HIC** | ✓ | ✓ | ✓ | ✓ |  | ✓ | ✓ | ✓ | ✓ | ✓ | ✓ | ✓ |  | ✓ | ✓ | (exact) |
| **Fiji** (Neal et al., 2020) | **UMIC** | ✓ | ✓ | ✓ | ✓ |  | ✓ | ✓ | ✓ | ✓ | ✓ | ✓ | ✓ |  | ✓ | ✓ | (exact) |
| **Hong Kong** (Kwok et al., 2014) | **HIC** | ✓ | 🗶 | ✓ | ✓ |  | ✓ | ✓ | ✓ | ✓ | ✓ | ✓ | ✓ |  | 🗶 | 🗶 |  |
| **Hong Kong** (Kwok et al., 2018) | **HIC** | ✓ | ✓ | 🗶 | 🗶 |  | ✓ | ✓ | ✓ | ✓ | ✓ | ✓ | ✓ |  | 🗶 | 🗶 |  |
| **Hong Kong** (Leung et al., 2017) | **HIC** | ✓ | ✓ | ✓ | ✓ |  | ✓ | ✓ | ✓ | ✓ | ✓ | ✓ | ✓ |  | ✓ | ✓ | (exact) |
| **India** (Kumar et al., 2018) | **LIC/LMIC** | ✓ | ✓ | ✓ | ✓ |  | ✓ | ✓ | ✓ | ✓ | ✓ | ✓ | ✓ |  | ✓ | ✓ | (exact) |
| **Kenya** (Kiti et al., 2014) | **LIC/LMIC** | ✓ | 🗶 | ✓ | 🗶 |  | ✓ | ✓ | ✓ | ✓ | ✓ | ✓ | ✓ |  | 🗶 | ✓ | (age groups) |
| **Peru** (Grijalva et al., 2015) | **UMIC** | ✓ | ✓ | ✓ | ✓ |  | ✓ | ✓ | ✓ | ✓ | ✓ | ✓ | ✓ |  | ✓ | ✓ | (exact) |
| **Russia** (Ajelli and Litvinova, 2017) | **UMIC** | ✓ | ✓ | 🗶 | 🗶 |  | ✓ | 🗶 | ✓ | ✓ | ✓ | ✓ | ✓ |  | 🗶 | ✓ | (exact) |
| **S Africa** (Dodd et al., 2015) | **UMIC** | ✓ | ✓ | 🗶 | ✓ |  | ✓ | ✓ | ✓ | ✓ | ✓ | ✓ | ✓ |  | ✓ | ✓ | (age groups) |
| **S Africa** (Wood et al., 2012) | **UMIC** | ✓ | ✓ | ✓ | ✓ |  | ✓ | ✓ | ✓ | 🗶 | ✓ | ✓ | ✓ |  | ✓ | ✓ | (exact) |
| **Senegal** (Potter et al., 2019) | **LIC/LMIC** | ✓ | ✓ | 🗶 | 🗶 |  | ✓ | ✓ | ✓ | ✓ | 🗶 | 🗶 | ✓ |  | 🗶 | 🗶 |  |
| **Thailand** (Mahikul et al., 2020) | **UMIC** | ✓ | ✓ | ✓ | 🗶 |  | ✓ | ✓ | ✓ | ✓ | ✓ | ✓ | ✓ |  | ✓ | ✓ | (age groups) |
| **Thailand** (Stein et al., 2014) | **UMIC** | ✓ | ✓ | 🗶 | 🗶 |  | ✓ | ✓ | ✓ | ✓ | 🗶 | 🗶 | ✓ |  | 🗶 | 🗶 |  |
| **Uganda** (le Polain de Waroux et al., 2018) | **LIC/LMIC** | ✓ | 🗶 | ✓ | ✓ |  | ✓ | ✓ | ✓ | ✓ | ✓ | ✓ | ✓ |  | 🗶 | ✓ | (age groups) |
| **Vietnam** (Horby et al., 2011) | **LIC/LMIC** | ✓ | ✓ | ✓ | ✓ |  | ✓ | ✓ | ✓ | ✓ | ✓ | ✓ | ✓ |  | ✓ | ✓ | (age groups) |
| **Zambia** (Dodd et al., 2015) | **LIC/LMIC** | ✓ | ✓ | 🗶 | ✓ |  | ✓ | ✓ | ✓ | ✓ | ✓ | ✓ | ✓ |  | ✓ | ✓ | (age groups) |
| **Zimbabwe** (Melegaro et al., 2017) | **LIC/LMIC** | ✓ | ✓ | ✓ | 🗶 |  | ✓ | ✓ | ✓ | ✓ | ✓ | ✓ | ✓ |  | ✓ | ✓ | (age groups) |
